# Empathy differences by gender and specialty preference in medical students: a study in Brazil

**DOI:** 10.5116/ijme.572f.115f

**Published:** 2016-05-21

**Authors:** Mariana A. Santos, Suely Grosseman, Thiago C. Morelli, Isabela C. B. Giuliano, Thomas R. Erdmann

**Affiliations:** 1 Family and Community Medicine Residency, Florianópolis Health Secretary, Santa Catarina, Brazil; 2 Department of Pediatrics, Medical School, Federal University of Santa Catarina, Brazil; 3 Department of Surgery, Medical School, Federal University of Santa Catarina, Brazil

**Keywords:** Medical Students, Empathy, Jefferson Scale of Empathy, gender differences, specialty interest, Brazil

## Abstract

**Objectives:**

We have conducted this study to assess medical students’ empathy and to examine empathy differences by students’ socio-demographic characteristics, including gender, and specialty preference.

**Methods:**

We have conducted a cross-sectional and descriptive research. Among 595 medical students registered at the Federal University of Santa Catarina (Brazil) in 2012, we have selected a sample of 320 enrolled in the first, third, fifth, seventh, ninth, eleventh, and in the last semester of the course. The response rate obtained was 70.6% (n=226). Data was collected by using a self-report questionnaire, and the variables analyzed included course semester, socio-demographic characteristics (such as age, gender, household monthly income and parents level of education), students’ specialty preference, and empathy assessed by the Jefferson Scale of Empathy. We have used descriptive statistics, 95% Confidence Interval for percentages, Student's t-test, and Analysis of Variance to analyze the data.

**Results:**

Mean empathy among students was (M=119.7, SD=9.9), with no difference by according to semester (F_(6,219)_=1.5, p=.2). Empathy means were higher among females (M=118.3, SD=10.6) than among males (M=121.0, SD=9.3, t_(222)_=-2.1, p=.032). Students who preferred a people-oriented specialty obtained significantly higher mean scores (M=121.5, SD=8.1) in comparison to students who preferred technology-oriented specialties (M=118.0, SD=11.3, t_(135)_=2.4, p=.02).

**Conclusions:**

Our study has found consistently high scores of empathy among medical students enrolled in all levels of training at the Federal University of Santa Catarina, and higher empathy among women and students who intend to pursue a people-oriented specialty. Conclusions on higher empathy among medical students require further study.

## Introduction

Empathy is an essential component of a physician’s therapeutic effectiveness and is associated with improved patient outcomes.^1^ Physician empathy increases rapport, patient confidence in the physician, and satisfaction with medical visits.^2^

A study conducted in 2009^3^ at two clinics in Wisconsin found that “clinical empathy, as perceived by patients with common cold, significantly predicts subsequent duration and severity of illness and is associated with immune system changes”. Another study conducted with patients seen at the clinic by doctors of the Department of Family and Community at Thomas Jefferson University found better clinical results in diabetic patients whose doctors were more empathic.^4^

The definition of empathy varies in the literature. According to Rogers^5^ empathy is the ability to put yourself in the other’s shoes, to see the world through their eyes and understand how they feel, without losing your own perspective. Morse et al^6^ consider empathy a construct composed of four dimensions. These dimensions are the emotional (intrinsic ability to understand the feelings), the moral (motivation to want to understand them), the cognitive (correctly understand the feelings), and the behavioral dimension (ability to communicate that one understands such feelings). Halpern^2^ considers that the function of empathy is not only to be aware of an emotional state, but also to know how it is to feel it. This author states that, to be empathic, the physician does not need to experience the patient’s emotions - which would be extremely exhausting - but to imagine how it is to feel them. The author mentions that, although you can list certain emotional behaviors and attitudes in a checklist to act out with the patient, it would be easier for the naturally empathic physician to demonstrate such behaviors and attitudes, and this may have a faster and more authentic therapeutic approach. For Hojat et al,^7^ the cognitive component predominates in Medical Empathy. They argue that empathy;

"is a predominantly cognitive attribute [...] that involves understanding [...] the patient’s experiences, concerns and the capability to communicate this understanding.[(...]."

Given the importance of empathy in medicine, Hojat et al,^8^, ^9^^, ^^10^ developed an instrument to assess it - the Jefferson Scale of Empathy (JSE), in order to address the lack of a specific empathy scale in healthcare research.

Several studies have used the JSE, and empathy scores have varied during undergraduate medical education, depending on the country where the study was being conducted. A decrease in empathy scores in clinical years was observed in the United States,^7^ while some studies in countries like Portugal,^11^^,^^12^ South Korea^13^ and Japan^14^^,^^15^ showed that empathy remained the same or even increased.

Taking into account that empathy is a crucial element of effective physician-patient communication and the need for nurturing empathy throughout medical training, it is important to study the characteristics of students associated with a greater empathy. Several studies worldwide have shown a greater empathy in women^11^^,^^14^^,^^17^^-^^25^ and among students who prefer a people-oriented specialty.^9^^,^^21^^,^^25^ A study in Kuwait found lower empathy among students with lower family income.^22^

Considering that cultural factors may influence empathy and its relationships, the purpose of this study was to assess medical students’ empathy and to examine empathy differences by students’ socio-demographic characteristics, including gender, and specialty preference.

## Methods

### Study design

This study was cross-sectional and descriptive.

### Participants and sample size

In Brazil, the medical course consists of 6 years, and clinical clerkships usually take place in the last 2 years of the course.

At the Federal University of Santa Catarina, where the study was conducted, the medical course is divided into 12 semesters, and student registration takes place twice a year, generally admitting 50 students per semester. This study was conducted in the second semester of 2012, when there was a total of 595 medical students enrolled at the Federal University of Santa Catarina.

Our sample comprised medical students from this University enrolled in the first, third, fifth, seventh, ninth, eleventh, and in the last semester of 2012. We’ve selected this sample in order to include the first semester students and assess differences that might take place each year. The last semester was included to assess empathy in students who were completing the course. There were 320 students enrolled in those semesters and a total of 226 agreed to participate, representing a 70.6% of response rate, being 47/51 of the 1st semester (92.1%), 36/45 of the 3rd semester (80%), 30/44 of the 5th semester (68.1%), 40/45, of the 7th semester (88.8%), 25/45 of the 9th semester (55.5%), 18/45 of the 11th semester (40%), and 30/45 of the 12th semester (66.6 %). The mean age of the 226 participants was 23.4 years old (SD = 3.5), of which 98 were males (43.8%, 95% CI: 40.5 - 47.1) and 126 were females (56.2%, 995% CI: 52.9 - 59.5).

Before gathering data, we submitted the research project to the Ethics Board of the “Governador Celso Ramos Hospital”, located in Florianópolis (Santa Catarina, Brazil), and it was approved under the protocol 2012/0019. We began gathering data after we received the approval of the Ethics Board. The participants of the study have provided informed consent, their anonymity was preserved, and the ethical regulations have been duly obeyed.

### Data collection

We used a self-report questionnaire to collect data. The variables analyzed included course semester, age, gender, skin color, religious belief, students’ city and state of birth, with whom they lived, household monthly income, highest level of education of each parent, students’ specialty preference, and empathy assessed by the Jefferson Scale of Empathy. This scale demonstrates consistent psychometric analysis (validity and reliability) and significant correlation with other measures aimed at human characteristics - as with the subscales "Empathic Concern" and "Perspective Taking" of the Interpersonal Reactivity Index (IRI).^26^ It can be applied to medical students, physicians, and other health care professionals. Because of its relevance, it has been translated into different languages for use in different countries. The JSE contains 20 items with statements that are answered in a 7-point scale Likert, where 1 is "strongly disagree" and 7, "strongly agree"; one half of the items are negative statements, and therefore, they are reversed for analysis.^8^ The score ranges from 20 to 140, with higher scores meaning a greater level of empathy.

The specialties were categorized according to people-oriented specialties (related to the general internal medicine, pediatrics and obstetrics and gynecology) and technology-oriented specialties (related to the surgical area and specialties such as ophthalmology, otorhinolaryngology, anesthesia, radiology, and pathology).^27^

In order to collect data, we’ve requested from the teachers of each semester forty-five minutes of their classes to conduct the research. Upon their agreement, we’ve visited the students in their classrooms, we’ve presented them with the study objective and procedures, and then, we’ve invited them to participate. We’ve provided the students who accepted to participate with informed consent, asking them to read it and sign it. Once the students completed their informed consent, we began applying the questionnaire.

### Data analysis

We analyzed data, using descriptive statistics, mean and Standard Deviation (SD). We’ve used the 95% Confidence Interval to compare the proportion of females and males in the sample; and we’ve used Student's t-test to compare means between two groups, and One-way between-groups Analysis of Variance (ANOVA) to compare means between more than two groups. The significance level was 95% (p <0.05).

We divided students’ age into two groups, using the mean age as cutoff point and we’ve divided household monthly income into two groups, using the 25 percentile as cutoff point between lower and higher incomes (R$5,000.00 which was equivalent to $2,380.00 in December of 2012).

## Results

Medical students’ mean empathy score on the JSE was 119.7 (SD=9.9), and their means in the first, third, fifth, seventh, ninth, eleventh, and last semester of the course were 121.7 (SD= 7.9), 116.4 (SD=10.3), 121.0 (SD=7.7), 120.1 (SD= 8.4), 117.0 (SD=12.1), 119.6 (SD=9.8), and 121.4 (SD=13.2), respectively. There were no significant differences between the groups (F_(__6, 219)_=1.5, p=.2].

When comparing empathy means by socio-demographic characteristics among the participants, the values found were: 118.2 (SD=10.6) among 98 males and 121.0 (SD=9.3) among 126 females, (t_(222)_=-2.1, p=.032, M=119.7, SD=8.5) among 105 students with ages equal or below 23.4, and 120.7 (SD=10.1) among 105 above this age group, (t_(208)_=-.78, p =.43,  M=119.8, SD=10.0) among 184 white (Caucasian) students and 119.6 (SD=9.5) among 42 non-white (non-Caucasian) students, (t_(224)_=.1, p=.92, M=120.9, SD=8.6) among 121 students who had religious beliefs and 118.3 (SD=11.2) among 103 who didn’t, (t_(189,3)_=-1.94, p=0.05, M=118.4, SD=9.5) among 61 students who were born in Florianópolis and 120.3 (SD=10.0) among 165 who were born in another city, (t_(224)_=-1.27, p=.77, M=119.2, SD=10.3) among 130 students who were born in Santa Catarina and 120.5 (SD=9.4) among 96 who were born outside the State of Santa Catarina, (t_(224)_=-1.0, p=.32, M=119.7, SD=9.9) among 174 students who lived with other persons and 119.8 (SD=10.2) among 52 who lived alone, (t_(224)_=-.062, p=.95, M=123.2, SD=6.7) among 30 students whose household monthly income was lower than or equal to R$5,000.00, and 119.5 (SD=10.3) among 125 with household monthly income above R$5,000.00; (t_(153)_=1.9, p=.06, M=120.0, SD=8.3) among 14 students whose parents’ level of education reached elementary school, 118.1 (SD=9.2) among 43 whose parents’ level of education reached high school; and 120.1 (SD=10.2) among 169 whose parents’ level of education was higher than the others mentioned before, (F_(2, .223)_=.70, p=.5).

Empathy mean score was 121.5 (SD=8.1) among 107 students who preferred a people-oriented specialty, and 118.0 (SD=11.3) among 79 who demonstrated preference towards a technology-oriented specialty, (t _(135.1)_ =2.4, p=.02).

## Discussion

We found that empathy scores remained high throughout the medical course of the University where the research was conducted. Some studies reported a decline in empathy scores during the clinical years.^7^^,^^22^^,^^24^^,^^28^^,^^29^ In the US, for example, this phase begins during the 3rd year of medical school (of a total of 4 years). Studies in other countries have not found a decline in empathy scores.^11^^-^^15^^,^^19^^,^^20^  In our medical school, early and consistent exposure to working with patients in community settings may help students to maintain higher levels of empathy, a conclusion partially supported by one of our previous studies.^30^

Higher empathy scores among the females analyzed in our study are consistent with other reports.^11^^,^^12^^,^^14^^,^^17^^-^^25^^,^^30^  Several authors have surmised hypotheses for this phenomenon.^12^^,^^19^^-^^21^^,^^31^ They include extrinsic factors (role expected by society) and intrinsic factors, such as biological characteristics - including correlation with neurological findings. Rueckert and Naybar^32^ found, exclusively in women, a correlation between the activation of the right cerebral hemisphere and empathy. Parental investment theory was also considered as an explanation to higher empathy scores among women. According to this theory, mothers are expected to develop a stronger sense of caring and to be more skilled in understanding their offspring emotions and needs in order to ensure their survival.^12^ In addition, studies conclude that women have a greater emotional receptivity and are more likely to develop and value interpersonal relationships and to offer more emotional support than men, tending to have more humanistic attitudes, greater social sensitivity and greater care. On the other hand, men would tend to adopt attitudes of "justice, independence and control".^12^ However, the finding of higher empathy scores among females who were medical students was not confirmed in studies in Portugal, South Korea, New Zealand and Iran,^13^^,^^15^^,^^28^^,^^33^ potentially indicating cultural influences on attitudes towards empathy.

Among those students in our sample who’ve indicated preference towards a specialty, those who preferred a people-oriented specialty had higher empathy scores than those who preferred a technology-oriented specialty, which is consistent with other studies.^7^^,^^21^^,^^25^ However, as Tavakol et al.^21^ point out, we cannot conclude that students with higher empathy scores would generally choose people-oriented specialties, since this question has not been rigorously studied.

We didn’t find differences between empathy scores and household monthly income or the level of parents’ education. Two studies report that lower class individuals respond to the environment with greater compassion34, generosity, trust, and cooperation.^35^ There are also studies linking social class to empathy. A study with a sample of university students and university workers applied three tests to evaluate the agreeableness and empathic accuracy (EA), which is the ability to infer accurately the emotions of others.^36^ The study has shown that women, people high in agreeableness, and less educated people had higher EA. In addition, lower class individuals have demonstrated more accuracy in the perception of emotions during social interactions; and the increased focus on the external social context explains this trend.^36^ There are probably cultural influences in the association of social class and empathy, though, as indicated by a contrary study from Kuwait.^22^

Religious belief was not associated with empathy scores in our study. Religion deals with themes that are relevant to medicine, such as human suffering and the dilemma of life and death, in addition to being associated with spirituality. In order to investigate the intensity of religious influence on attitudes toward patients, a study applied two questionnaires to 324 doctors working in public hospitals in Poland.^37^ There was a statistically significant difference between the degree of religiosity between surgical and non-surgical specialties, favoring the former. There was also a significant positive correlation between a religiosity scale and dimensions of holism, empathy, and altruism. As for the questions about religious faith, most agreed that this attribute enables them to better cope with professional obligations, and that the awareness of God's presence helps in difficult situations. Although our study has not found this association, the finding of the aforementioned study showed that socio-cultural characteristics of physicians could influence attitudes towards patients. These characteristics cannot be ignored and should be further investigated.

This study may reveal limited conclusions due to the fact that it was conducted in just one university. Its cross-sectional design and the possibility of social desirability bias may also have influenced the answers obtained through the questionnaire. Albeit in a country where different cultures should not be overlooked, the association between higher empathy among women and among students with a preference towards people-oriented specialties was confirmed.

## Conclusions

Our study found consistently high scores of empathy in medical students enrolled in all levels of training at the Federal University of Santa Catarina, Brazil, as well as higher empathy in women and students who intend to pursue people-oriented specialties. Conclusions on higher empathy among medical students require further study.

### Acknowledgements

We sincerely thank the students who have participated in this research. We are grateful to Professor Dennis Novack, for his kind and in-depth revision of the paper.

### Conflict of Interest

The authors declare that they have no conflict of interest.
